# A conceptual model for understanding post-release opioid-related overdose risk

**DOI:** 10.1186/s13722-019-0145-5

**Published:** 2019-04-15

**Authors:** Paul J. Joudrey, Maria R. Khan, Emily A. Wang, Joy D. Scheidell, E. Jennifer Edelman, D. Keith McInnes, Aaron D. Fox

**Affiliations:** 1 VA Connecticut Healthcare System, West Haven Campus, 950 Campbell Ave, West Haven, CT 06516 USA; 2 National Clinician Scholars Program, Yale School of Medicine, 333 Cedar Street, Sterling Hall of Medicine IE-68, PO Box 208088, New Haven, CT 06520 USA; 3 Department of Population Health, New York University, 227 East 30th Street, New York, NY 10016 USA; 4 Department of Internal Medicine, Yale School of Medicine, Yale University, 367 Cedar Street, New Haven, CT USA; 5 Department of Veterans Affairs, Center for Healthcare Outcomes and Implementation Research, Edith Nourse Rogers VA Hospital, Bedford, MA USA; 6 Department of Health Law Policy and Management, Boston University School of Public Health, Boston, MA USA; 7 Albert Einstein College of Medicine, Bronx, NY 10461 USA; 8 Montefiore Medical Center, Bronx, NY 10467 USA

**Keywords:** Criminal justice system, Opioid-related overdose, Mortality, Conceptual model

## Abstract

Post-release opioid-related overdose mortality is the leading cause of death among people released from jails or prisons (PRJP). Informed by the proximate determinants framework, this paper presents the Post-Release Opioid-Related Overdose Risk Model. It explores the underlying, intermediate, proximate and biological determinants which contribute to risk of post-release opioid-related overdose mortality. PRJP share the underlying exposure of incarceration and the increased prevalence of several moderators (chronic pain, HIV infection, trauma, race, and suicidality) of the risk of opioid-related overdose. Intermediate determinants following release from the criminal justice system include disruption of social networks, interruptions in medical care, poverty, and stigma which exacerbate underlying, and highly prevalent, substance use and mental health disorders. Subsequent proximate determinants include interruptions in substance use treatment, including access to medications for opioid use disorder, polypharmacy, polydrug use, insufficient naloxone access, and a return to solitary opioid use. This leads to the final biological determinant of reduced respiratory tolerance and finally opioid-related overdose mortality. Mitigating the risk of opioid-related overdose mortality among PRJP will require improved coordination across criminal justice, health, and community organizations to reduce barriers to social services, ensure access to health insurance, and reduce interruptions in care continuity and reduce stigma. Healthcare services and harm reduction strategies, such as safe injection sites, should be tailored to the needs of PRJP. Expanding access to opioid agonist therapy and naloxone around the post-release period could reduce overdose deaths. Programs are also needed to divert individuals with substance use disorder away from the criminal justice system and into treatment and social services, preventing incarceration exposure.

## Introduction

The United States has high rates of incarceration and opioid-related overdose mortality. Starting in the 1970s, the United States incarceration rate quadrupled over the ensuing four decades such that in 2016, 2.3 million individuals were involved with the criminal justice system [1]. Increased criminal penalties and prosecution of drug-related crimes contributed to mass incarceration’s rise and exacerbated racial disparities within the criminal justice system [2, 3]. Meanwhile, starting in the 1990s, opioid use, opioid use disorder, and overdose also skyrocketed with a disproportionate impact on people released from jail or prison (PRJP) [3, 4]. Between the year 2000 and 2014, the United States experienced a 137% increase in the rate of drug overdose deaths and 200% increase in opioid-related overdose mortality [5, 6]. Opioid-related overdose mortality continues to increase, with 33,091 opioid-related overdose fatalities occurring within the United States in 2015 as heroin and synthetic opioid use increases [7, 8]. Up to 20% of individuals housed within prison in the United States meet criteria for opioid use disorder (OUD) [9, 10]. In 2016, at least 20% of people with OUD had experienced criminal justice involvement in the prior year [11]. The problems of mass incarceration and opioid overdose are clearly interrelated.

For PRJP, the community re-entry period starts upon release from jail or prison and extends beyond the first year following release. Over a decade of scholarly work demonstrates that PRJP are particularly vulnerable to post-release opioid-related overdose mortality [12–19]. Drug overdose is the leading cause of death following release from the criminal justice system internationally; the majority of overdose deaths are opioid related [16, 18–21]. A seminal study in the United States demonstrated that after controlling for demographic factors, individuals released from prison in Washington State had 129 times greater risk of drug overdose in the first 2 weeks post-release relative to the general population. The majority of these overdoses involved opioids [15–17]. Elevations in overdose risk have been consistent internationally, among diverse demographic groups, and whether release was from long-term prison or shorter jail stays [16].

The intertwined epidemics of mass incarceration and opioid overdose create a complex risk environment where environmental, social, and biologic factors influence post-release opioid-related overdose mortality. Identifying factors that mediate and modify post-release opioid-related overdose mortality risk can create opportunities for novel interventions and programs. Previous reviews and theoretical models of opioid-related overdose have examined the biological determinants or mechanisms of overdose in the general population [22–24]. However, underlying environmental and social factors that contribute to post-release opioid-related overdose mortality in correctional populations are likely different and have not been systematically explored [25]. Given the potential number of factors, their interactions, and the need for institutional changes to address the risk environment, a common comprehensive model that explains post-release opioid-related overdose mortality is needed to direct intervention design and broad criminal justice reform efforts in this historically neglected population.

**Fig. 1:**
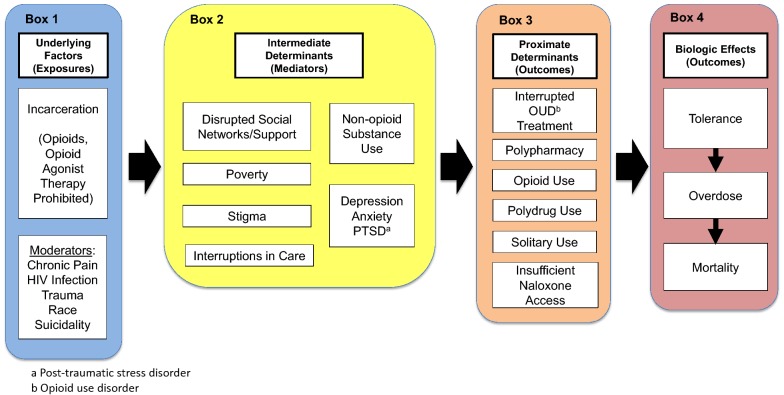
The post-release opioid-related overdose risk model: a conceptual model of the putative mechanisms behind post-release opioid-related overdose mortality

To address this need, we developed a conceptual model of the putative mechanisms contributing to post-release opioid-related overdose mortality. This article will review existing literature on the known risk factors underlying post-release opioid-related overdose mortality. We organize these factors within a heuristic model (Fig. 1) which includes: underlying factors and setting, intermediate determinants, proximate determinants, and biologic effects. Finally, we will present how our model can inform policy and future research directed at reducing opioid-related overdose mortality among PRJP.

## Development of post-release opioid-related risk model

The proposed conceptual model, the Post-Release Opioid-Related Overdose Risk Model, is a modified version of the proximate determinants framework. The proximate determinants framework describes demographic and epidemiologic factors which act as a “hinge” connecting social factors with the biologic events [26, 27]. Adaptations of the framework have used common elements indicating that the underlying factors, proximate determinants, and biological determinants jointly determine the probability of the health outcome of interest [28, 29].

The Post-Release Opioid-Related Overdose Risk Model describes pathways that lead to elevated opioid-related overdose risk after release from incarceration, considering risk factors for overdose that are common among PRJP and the unique experience of criminal justice involvement itself (incarceration, release and community re-entry). In the model, incarceration is considered an “underlying factor” or adverse exposure that affects opioid-related overdose risk. The effect is moderated by the greater prevalence of sociodemographic or clinical factors (e.g., chronic pain, HIV-infection, prior trauma, race, and suicidality) associated with incarceration. The effect of incarceration is mediated through “intermediate determinants,” which result from incarceration and increased risk of opioid-related overdose (e.g., social network disruption, poverty, stigma, alcohol and drug use). Intermediate determinants do not directly lead to opioid-related overdose mortality; however, they are causally related to post-release opioid-related overdose mortality through the “proximate determinants” of opioid-related overdose and in turn biological effects. The proximate determinants of overdose risk (e.g., lack of access to care and treatment, opioid use, solitary use, and barriers to naloxone access) are factors that connect the experience of incarceration with biologic outcomes. Proximate determinants are directly related to the outcomes of overdose and mortality through the “biologic effects” (i.e., reduced tolerance to opioids and respiratory depression).

To develop the Post-Release Opioid-Related Overdose Risk Model, we brought together a team of researchers and clinicians with expertise relating to the criminal justice system and associated fields, including addiction medicine, HIV medicine, and chronic pain. The model and associated literature review were developed jointly using an iterative process. First, an initial model of proposed risk factors was developed via group discussion and consensus. Then members of our research team submitted literature relevant to each model risk factor and pathway and this literature was then supplemented by searches of MEDLINE and Google Scholar databases. Informed by this literature, we then revised our model and identified risk factors and model pathways requiring further literature searches. Several cycles of model revision and narrative literature review were pursued, from October 2017 to July of 2018, until the group agreed saturation of pathway relevant literature was achieved or a gap in knowledge was identified and a final model was agreed upon. In the next sections the major model components are discussed, starting at the left, with underlying factors (Box 1), and moving to the right through intermediate determinants (Box 2) and proximate determinants (Box 3) to biologic effects (Box 4).

## Underlying factors and setting

All post-release opioid-related overdose deaths share the common exposure of time within the criminal justice system. The underlying conditions of the criminal justice system influence subsequent opioid-related overdose risk. PRJP have high rates of chronic diseases [25], and they have worse health outcomes than populations without criminal justice involvement. Chronic pain, HIV, and trauma, all prevalent among PRJP, may be linked to opioid-related overdose (Fig. 1, Box 1). Pathways connecting incarceration to opioid-related overdose are likely different for sub-groups with (versus without) these conditions. Comprehensive efforts to reduce post-release opioid-related overdose mortality will need to address these underlying factors.

### Chronic pain

PRJP have high rates of chronic pain which may increase utilization of acute medical services and exposure to prescription opioids before and during incarceration. Uncontrolled pain is a common reason for prescription opioid misuse [30]. Among all adults, an initial opioid prescription as short as 6 days was associated with an increased probability of long term opioid use [31]. Higher doses of opioid therapy for acute and chronic non-cancer pain was associated with opioid-related overdose risk [32]. Among individuals within a county jail over the age of 55, 75% reported a pain related problem and 39% reported severe frequent pain. Of those reporting severe frequent pain, 70% had received a prescription opioid from a correctional provider within 1 week of interview [33]. This suggests acute and chronic pain among adult individuals with justice involvement, if managed equivalently to community settings, may similarly increase opioid exposure, long-term opioid use, and potentially opioid-related overdose risk. Chronic pain may also affect mental health and substance use among PRJP, increasing the risk of post-release opioid-related overdose. In populations without criminal justice involvement, chronic pain is associated with increased odds of mood, anxiety and substance use disorders [34].

### HIV

The prevalence of HIV-infection is higher among PRJP than the general population, which may also interact with factors associated with post-release risk of opioid-related overdose. Among adults continuously housed within the criminal justice system the mean baseline prevalence of HIV is 2.1%, but among individuals released and re-incarcerated the mean prevalence is 6.1%. The mean HIV prevalence is also elevated among men who have sex with men (6.1%) and people who inject drugs (18.5%) within criminal justice settings [35]. Within the general population, HIV seropositivity is associated with an increased risk of drug overdose [36]. Among veterans, receipt of long-term opioids was independently associated with increased risk of mortality, especially among patients living with HIV compared to those without HIV [adjusted hazard ratio (95% CI) 1.54 (1.21, 1.96) vs. 1.35 (1.14, 1.61)] [37]. HIV infection and incarceration may interact to augment the underlying pathways leading to opioid-related overdose through cumulative disadvantage. Individuals living with HIV and with a recent history of justice involvement were more likely to be homeless, unemployed and previously diagnosed with a mental illness relative to those with a recent history of justice involvement without HIV [38]. Among people who inject drugs living with HIV, those with recent criminal justice exposure had 25% greater adjusted odds of lapses in medical care relative to those without criminal justice exposure [39]. Having multiple stigmatized identities may be particularly difficult for PRJP and may influence whether they seek medical care post-release.

### Trauma

The increased prevalence of trauma among PRJP may increase the risk of post-release opioid-related overdose mortality. PRJP report a history of physical assault at rates 13–27 times greater than the general population [40]. Among a national sample, 48% of women released from jail or prison reported being physically or sexually abused prior to incarceration and 27% reported having been raped [41]. Previous research has demonstrated an association between a history of trauma and increased risk of opioid-related overdose. Physical or sexual violence was associated with, respectively, 36% and 48% greater odds of a non-fatal overdose event among people who inject drugs [42]. Removal from one’s family as a child was associated with increased odds of post-release non-fatal overdose events among people who inject drugs who were recently released from prison [43]. The high prevalence of trauma among PRJP may interact with other factors mediating opioid-related overdose mortality. In a national sample of non-institutionalized men and women, increasing exposure to violence was associated with increasing rates of polypharmacy, including antidepressants, tranquilizers, and analgesics [44]. Within a nationally representative non-institutionalized sample of women, those with life history of post-traumatic stress disorder or history of drug or alcohol facilitated rape were more likely to report non-medical use of prescription drugs [45].

### Race

Within the United States, blacks are disproportionately represented within jails and prisons relative to whites [2]. Since the 1980s, blacks have been incarcerated at rates five to seven times greater than whites [46]. Upon release from jail or prison, blacks face greater stigma and discrimination relative to whites, which may affect employment and access to medical care. [47, 48]. This type of discrimination may affect the risk of post-release opioid-related overdose; however, post-release opioid-related overdose mortality is greater among whites released from jail or prison relative to blacks and other racial minorities [15, 16]. Understanding how race mediates access to medical care, receipt of opioid analgesics or opioid use disorder treatments, and other factors that influence post-release opioid-related overdose mortality will be essential for efforts to reduce overdose risk for all PRJP.

### Suicidality

PRJP have an underlying elevated risk of suicide, which also may affect post-release opioid-related overdose risk. In criminal justice populations, many risk factors for overdose and suicide are similar [49]. Men released from jail or prison have a six-fold increased risk of suicide-related mortality relative to the general population and for females release from jail or prison the risk is even greater [40]. Among non-institutionalized adults, prior suicide attempts are associated with non-fatal overdose [50]. Like overdose risk, suicide risk increases post-release, most likely because of the extreme stressors of community re-entry [51, 52]. It can be difficult to distinguish intentional and accidental opioid-related overdose events, and intentional overdose deaths may be under-reported [53]. This under reporting suggests suicide may play an underappreciated role in post-release opioid-related overdose mortality.

## Intermediate determinants

During community re-entry, the risk of post-release opioid-related overdose mortality is increased through the intermediate determinants of disrupted social networks/support, poverty, interruptions in health care access, stigma, and an exacerbation of underlying psychiatric and substance use disorders (Fig. 1, Box 2) [54, 55]. Incarceration exposure leads to these intermediate determinants and efforts to successfully mitigate the risk of post-release opioid-related overdose could target these factors to reduce their influence on subsequent proximate and biological determinants.

### Disrupted social networks

Social support buffers the negative health effects of stressful events, such as incarceration, and can promote healthy behaviors [56]. The process of incarceration physically removes a person from their family, friends, and community, interrupting social relationships during a period of increased stress. Between 50 and 80% of individuals are in committed relationships at the time of prison entry, but between 30 and 50% of those relationships end during incarceration [57, 58]. The loss of a committed partner during incarceration is linked to increased post-release stress and substance use compared to men who remained with a committed partner. For example, in a sample of African American men recently released in North Carolina, those whose committed relationships ended during incarceration had greater stress associated with re-entry when compared to men who remained in a relationship with a committed partner [59]. Within this population, incarceration-related partnership disruption independently predicted post-release binge drinking. Upon release, criminal justice exposure may continue to indirectly disrupt social supports. Males released from jail or prison in Ohio reported using strategies of “preventative” social withdrawal and secrecy to avoid anticipated discrimination [60]. In addition, individuals with a recent history of justice involvement may avoid reintegration into the community to avoid re-exposure to their prior lifestyle, leading to isolation at home and avoidance of old contacts. Qualitative interviews with PRJP indicate the importance of social support as a protective factor against returning to drug use and overdose during re-entry [61].

### Poverty

Incarceration also has the long-term, often unintended, consequence of trapping individuals in poverty upon transitioning to the community. Once released, PRJP—particularly those convicted of felonies and those on sex offender registries—are no longer eligible for specific educational, employment, or housing opportunities [62]. Some housing policies may exclude PRJP, potentially leading to housing instability and homelessness [63.]. In the Fragile Families study, PRJP had four times the odds of homelessness, and incarceration was associated with increased risk of eviction for those living in public housing [63]. Housing insecurity is tied to labor market potential, which is also negatively affected by incarceration [64]. Incarceration limits employment opportunities by limiting access to education and/or eligibility for government jobs and professional licenses [65]. Employers may be less likely to hire those with prior criminal justice exposure. Among generic job applications submitted to low wage jobs in New York city, PRJP were half as likely to be called back or receive a job offer relative to those without a criminal record and this disparity was more pronounced among blacks relative to whites [48, 66]. These barriers make employment difficult to attain after incarceration. In a longitudinal study of PRJP in Ohio, Texas, and Illinois, less than half were currently employed 8 months after release and their median monthly income was approximately $700, which equates to $8.95 per hour [67]. The stress due to unmet financial needs may drive PRJP to use substances to cope. In a sample of individuals with a history of substance use recently released from correctional facilities, those experiencing unstable housing reported the highest levels of drug use [68].

### Stigma

The problems of social isolation and poverty are further exacerbated by incarceration-related stigma. Stigma is described as unfavorable attitudes, beliefs, and policies directed toward people perceived to belong to an undesirable group. There are few groups as highly stigmatized as PRJP [69]. PRJP perceive high levels of stigma, which may lead them to internalize the stigma and ultimately self-stigmatize [47, 70]. Among PRJP in New York state, 65.3% reported discrimination due to their prior criminal justice involvement [71]. In comparison to college students, PRJP perceived more stigma in the general public regarding incarceration [70]. Stigma impacts post-release success among PRJP, including gaining employment and risk of recidivism [70]. In a sample of women released from jail or prison with a history of substance use, stigma was highlighted as a factor impacting all aspects of community re-entry, including basic survival, access to treatment, and family reintegration [72]. Stigma is linked to poor psychological functioning, such as increased depressive symptoms and to substance use [73, 74]. Further, individuals who feel stigmatized, especially within health care settings, may avoid treatment and health care except in the case of emergencies. Among adults in the community who are living with HIV and inject drugs, those who reported (versus did not report) internalized HIV or substance use-related stigma had lower odds of health service utilization [75]. In a sample of transgender men in the community, those who experienced stigma from healthcare providers had increased risk of using drugs to cope with the mistreatment [76]. Hence, incarceration-related stigma may exacerbate post-release psychopathology, which in turn, can lead to increased opioid use and overdose mortality risk.

### Interruptions in care

After incarceration post-release interruptions in health care are common [77–80]. Therefore, PRJP are less likely than the general population to have a primary care physician and more likely to use emergency departments or experience preventable hospital admissions [79, 81, 82]. Among a group of men released from jail or prison with chronic health conditions, barriers to accessing clinical care included lack of insurance, stigmatization, substance use, being on parole, institutional bureaucracy, and being assigned to the indigent system. These men reported reducing their utilization of the healthcare system due to these barriers [83]. Reduced access to care has implications for screening to identify overdose risk and interruptions in access to medications for opioid use disorder (MOUD). More than 1 in 15 adults released from jail or prison were taking a prescription medication at the time of incarceration and 41.8% stopped taking these medications following incarceration [84]. In addition, psychiatric medication regimens disrupted during incarceration and barriers to care after release hinder the continuity of mental health care [85]. This may result in under-treatment of symptoms in some cases and over-sedation in other cases [86–89]. Post-release changes in mental health treatment may lead to polypharmacy such as use of opioids with benzodiazepines, especially in the context of treating post-release anxiety disorders; polypharmacy use is a strong overdose risk factor [90, 91].

### Non-opioid substance use

Unhealthy alcohol use and injection drug use are prevalent among PRJP. Among PRJP, rates of alcohol use disorder ranged from 18 to 30% among men and 10–24% among women. Rates of drug use disorders among men ranged 10–48% and for women 30–60% [10]. Upon release, the stress of re-entry may exacerbate substance use disorders. Among PRJP, problems with family, friends, and significant others were associated with 3 times the odds of substance use and over 2.5 times the odds of unhealthy alcohol use [92]. Women with a history of justice involvement report drug and incarceration related stigma contributed to substance use relapse and recidivism following re-entry [72]. Non-opioid substance use may increase the risk of post-release opioid-related overdose mortality. Among adults in the community, alcohol was involved in over one-fifth of prescription opioid-related overdose deaths [93].

### Depression, anxiety, and post-traumatic stress disorder

PRJP also have high prevalence of psychiatric disorders, including depression, anxiety and post-traumatic stress disorder, which may increase the risk of post-release opioid-related overdose [40]. Estimates suggest 50–60% have a mental health disorder including 20–30% with symptoms of major depression [40, 94, 95], and between 40 and 50% exhibit both psychiatric and substance use disorders [96]. High levels of psychiatric symptoms among PRJP may increase exposure to other risk factors for post-release opioid-related overdose including prescription opioids [97, 98], benzodiazepines [99], and alcohol [98, 100–102]. Among adults receiving long-term opioid therapy for chronic pain in the community, those with moderate and severe depression were 1.8 and 2.4 times more likely to report misuse of opioids for non-pain symptoms [98].

Criminal justice exposure itself may exacerbate underlying psychiatric disorders. The stressful and disruptive nature of incarceration and release appear to underlie post-release increases in psychiatric symptoms [103]. PRJP with (vs. without) a history of exposure to solitary confinement had nearly fourfold increased odds of positive post-traumatic stress disorder screen at the time of first post-release primary care contact [104]. People released from the New York City jail system who had been assigned to solitary confinement were 3.2 times more likely to commit an act of self-harm compared to those without solitary confinement exposure. While only 7.3% of people released from jail received any solitary confinement, 53.3% of self-harm and 45.0% of potentially fatal self-harm occurred within this group [105]. While a substantial proportion of PRJP have histories of psychiatric disorders at the time of incarceration, the experiences of detention and release may also exacerbate symptoms; some evidence indicates acute effects immediately following release from prison and other studies suggesting long-term post-release psychiatric symptoms [106]. In a sample of individuals being released from incarceration in Rhode Island, one-third had worse depression symptoms upon return to the community [103]. PRJP may self-medicate with substance use as a means of coping with psychiatric disorder symptoms upon release [61], and post-release anxiety treatment with benzodiazepines can increase polydrug use and risk of opioid-related overdose [85].

## Proximate determinants

While intermediate determinants highlight the indirect impact of incarceration, proximate determinants directly influence post-release opioid-related overdose risk (Fig. 1, Box 3). These proximate determinants include opioid use, interrupted opioid use disorder treatment, polydrug use, solitary substance use, polypharmacy, and insufficient naloxone access.

### Opioid use

Some individuals use illicit opioids throughout their incarceration, and even those who maintain abstinence during incarceration may start again post-release [107]. Upon release from jail or prison, the intermediate determinants of disrupted social networks, poverty, stigma, and exacerbation of mental health and other substance use disorders increases the risk of returning to opioid use post-release [61, 68, 72, 98]. The risk of returning to opioid use following release from jail or prison may be modified by community corrections programs (parole and probation) which present an opportunity to screen and monitor for opioid use and OUD [108].

### Interrupted opioid use disorder treatment

Lack of MOUD availability during incarceration creates an interruption in care affecting the post-release period. MOUD includes opioid agonist therapy (OAT)—methadone and buprenorphine—and the opioid antagonist naltrexone. Together they are the mainstay of treatment for OUD and are broadly recommended by numerous professional organizations and guidelines [109–113]. Research demonstrates OAT reduces overdose and mortality risk [114–116]. Naltrexone can reduce the risk of post-release opioid relapse, but its effect on opioid-related overdose mortality among PRJP is less clear [117–119]. Despite strong consensus on the effectiveness of OAT, access to OAT among those involved with the justice system remains low, with many corrections systems prohibiting access to these potentially lifesaving medications [120, 121]. People receiving OAT in the community typically have their medication withdrawn during incarceration, and for those not enrolled in treatment, OAT are rarely started during incarceration or at the time of release [122]. In 2014, only 4.6% of individuals within the criminal justice system, including community corrections, referred for treatment of OUD received either methadone or buprenorphine therapy compared to 40.9% of individuals referred outside of the criminal justice system [123]. Access to OAT is particularly poor in jails where less than 1% of individuals needing methadone receive the therapy [124]. While 55% of prison systems report offering methadone, over half of these only offer methadone to individuals who are pregnant or diagnosed with chronic pain [120]. PRJP participating in community corrections programs may continue to face barriers to OAT. Many community corrections agents report little autonomy to refer individuals to OAT and some worry about the abuse potential of the medications [125].

Data from England and Australia demonstrate that individuals with OUD who leave jail or prison receiving an effective dose of OAT are much less likely to die of an overdose than those who are not receiving treatment [126, 127]. Detailed interviews with PRJP also suggest that the experiences of opioid withdrawal during incarceration after OAT cessation may dissuade them from restarting OAT following release [128–130]. During community reentry, exposure to illicit substance use is common, often triggering opioid relapse, while enrolling in OUD treatment is hindered by other intermediate determinants, including health insurance termination, emotional or psychological distress, and competing priorities, such as securing housing or employment [61, 107, 131]. Therefore, incarceration can prevent MOUD access both during incarceration, as well as upon release due to negative experiences. Incarceration is also a missed opportunity to initiate OAT, which could increase treatment utilization post-release and potentially prevent opioid-related overdoses [132–135].

### Polypharmacy

PRJP, which have high prevalence of chronic mental health conditions [40], are also frequently prescribed sedating medications that may contribute to overdose risk. Adults released from jail or prison within the United States are more likely to be prescribed antipsychotic drugs, such as aripiprazole and quetiapine, than commercially insured adults [136]. Among PRJP in East England, psychotropic medication prescribing was 5.5 times higher among men and 5.9 times higher among women relative to community prescribing rates after adjusting for age. When sedating medications, especially benzodiazepines or multiple medications, are combined with opioids, overdose risk increases [137, 138]. In another cohort of people supervised by a community corrections program in Alabama, more than 10% had both opioids and benzodiazepines present upon urine drug testing, and concurrent use was associated with having a drug-related offense [139]. The underlying increased prevalence of risk factors and chronic conditions such as chronic pain, HIV, and exposure to trauma may also increase health care use during incarceration, which creates additional opportunities for polypharmacy. People in prison within the United Kingdom consulted primary care doctors three times more frequently than community populations after controlling for demographic characteristics [140].

### Polydrug use

Mixing illicit substances is another risk-factor for post-release opioid-related overdose that is common among PRJP. In a Canadian cohort of people who use drugs, those with (vs. without) a recent history of justice involvement had two times greater odds of reporting a nonfatal overdose, and daily heroin, cocaine, methamphetamine, and benzodiazepine use were all associated in multivariable analysis with nonfatal overdose [141].

### Solitary use

Another risk factor for post-release opioid-related overdose is using alone, because if someone overdoses while alone, there may be no one there to provide assistance, administer naloxone, or call for help. In examining heroin-related overdose deaths in San Francisco from 1997 to 2000, researchers estimated that 68% of deaths occurred when the overdose victim was alone [142]. Another cohort study from five large United States cities reported that 15% of adults who injected illicit drugs always injected alone, and in multivariable analysis, having spent time in jail was associated with always injecting alone [143]. People who inject drugs may start with low levels of social support and then incarceration can distance them from their friends and families [61]. Disrupting social networks could also lead PRJP to buy illicit opioids from new unfamiliar sources when they return the community, which could then increase overdose risk by increasing unintentional exposure to potent synthetic opioids, like fentanyl [144].

### Insufficient naloxone access

There have been few efforts to target PRJP for overdose prevention interventions, such as naloxone training and distribution. Large-scale distribution of naloxone to individuals being released from prison is feasible and acceptable [145]. Naloxone training appears to be effective in increasing incarcerated individuals’ knowledge about naloxone and its use [146]. In New York, a pilot program trained 700 people in prison to administer naloxone to reverse opioid-related overdose, but only 200 received take-home naloxone kits at the time of release [147]. Potential barriers to implementing take-home naloxone in correctional facilities include: misinformation about naloxone, difficulty identifying and engaging people at risk for opioid-related overdose, and the need for senior administrative support for program implementation [148]. Despite the logistical challenges in implementing overdose prevention programs, this remains a promising strategy to reduce the risks of opioid-related overdose immediately post-release.

## Biologic effects

The pathophysiology of opioid-related overdose mortality is well understood. Opioids suppress respiratory drive and this physiology suggests how interruptions in opioid use can acutely increase the risk of fatal overdose (Fig. 1, Box 4). Tolerance to the euphoric effects of opioids with repeated use leads to an escalation of dose, while any voluntary or involuntary abstinence causes a rapid loss of respiratory tolerance. Individuals who suddenly return to an opioid dose that previously produced euphoric effects without dangerous levels of respiratory depression, may overdose due to the absence of this protective respiratory tolerance [22]. This physiology means lower opioid doses may result in overdose mortality among people returning to use after a period of abstinence, and previous research supports this mechanism. A post mortem analysis of morphine hair content among people who use heroin and experienced a fatal overdose, found people abstinent from use prior to overdose had lower morphine levels relative to people actively using heroin [149].

The overall setting, intermediate, and proximate conditions faced by PRJP act to reduce opioid tolerance. Denied access to OAT while incarcerated, individuals lack tolerance to the respiratory effects of opioids upon release. Back within the setting of their previous use following the additional exposure of incarceration, individuals face barriers to engage with addiction treatment services and initiate protective OAT. Many individuals return to opioid use in these circumstances and experience a fatal overdose. Receipt of buprenorphine or methadone during incarceration prevents the loss of respiratory tolerance and reduces opioid-related mortality post-release [127].

In recent years, the increase in prevalence of synthetic opioids, such are fentanyl, has added an additional risk to post-release opioid use. Fentanyl is 50–100 times more potent than morphine, and fentanyl and other synthetic opioids are often mixed with heroin, cocaine, or other compounds and sold to individuals who may lack knowledge of the contents [150]. This uncertainty increases the risk of overdose with any use of illicit opioids, even among experienced users. From 2013 to 2014, the age adjusted rate of synthetic opioid (fentanyl and tramadol) related overdose mortality increased by 80% [151]. Research suggests the increase in synthetic opioid-related overdose mortality does extend to PRJP. Among PRJP within in the past year in Rhode Island, the risk of fentanyl related overdose nearly doubled from 2014 to 2015 [144].

## Policy and research implications

Given the societal forces that underlie mass incarceration and the opioid epidemic, the high prevalence of OUD and criminal justice exposure is likely to continue in the near future. Thus, interventions are urgently needed to mitigate post-release opioid-related mortality risk. Two interventions, expanded access to OAT during and after incarceration and expanded access to naloxone upon release, are specific interventions that act on key mediators of opioid-related overdose and could reduce mortality in post-release populations. Federal, state, and local jurisdictions should adopt policies that require MOUD access within all jails and prisons for those with medical indication. Lessons learned from existing OAT programs within criminal justice settings, should be widely disseminated, and additional research should establish best practices (Table 1). Additionally, expansion of access to MOUD in community settings is also needed, including access to buprenorphine and methadone within community clinics and emergency departments where PRJP are likely to seek treatment.

**Table 1:** Post-release opioid-related overdose mortality: areas of further research

Topic	Area of inquiry
*Investigation of mechanisms*	
Chronic pain	Prevalence and self-medication hypothesis
HIV	Rate of post-release opioid-related overdose and living with HIV
Trauma	Rate of post-release opioid-related overdose and trauma exposure
Race	Race and opioid use disorder treatment and OAT access
Suicidality	Rate of post-release opioid-related overdose and suicidality
Mental health (depression, anxiety, and PTSD^b^)	Prevalence pre and post-release
Non-opioid substance use	Prevalence pre and post-release
	Intermediate determinants associated with substance use disorders
Disrupted social networks	Interventions to assist re-integration during the post release period
Poverty	Post-release poverty and mental health and substance use
Polydrug use	Prevalence and independent effect of CJ^a^ exposure on the risk of polysubstance use
Polypharmacy	Relationship between pre and post-release prescribing of sedating medications
Effect of probation or parole	Independent effect of probation and parole on post-release opioid related overdose mortality
Type of criminal charge or conviction	Relation to stigma and post-release opioid-related overdose risk
Relationships between risk factors	Investigation of the mediating or moderating relationships between risk factors
*Investigation of novel interventions*	
Chronic pain	Non-opioid pain management within jail or prison
Race	Reduction of criminal justice exposure among blacks
Interruptions in care	Improve engagement with healthcare services post-release
Solitary use	Safe inject sites to reduces rates of solitary use post-release
Care coordination	Integrate CJ, healthcare, and community organizations
Naltrexone	Effectiveness of reducing opioid-related overdose mortality
New formulations of medications	Efficacy and effectiveness of extended release buprenorphine
Police diversion programs and drug courts	Process evaluation and effectiveness at reducing criminal justice exposure and opioid-related overdose
*Implementation and dissemination*	
Interrupted opioid use disorder treatment and OAT^c^ access	OAT programs pre and post-release
Interrupted naloxone access	Naloxone program pre and post-release

^a^Criminal justice

^b^Post-traumatic stress disorder

^c^Opioid agonist therapy

Similarly, prisons and jails should be required to provide naloxone training and take-home kits to all individuals transitioning out of the criminal justice system with an elevated risk of opioid-related overdose. At-risk populations include people with current or past opioid use disorder, and people prescribed long-term opioid therapy for pain. Evidence-based dissemination and implementation interventions are needed (Table 1) to improve the distribution and utilization of naloxone during the immediate post-release periods and when risk of overdose mortality is greatest.

While expanded OAT and naloxone access are interventions ready for dissemination, other targets within our risk model will require additional work. The complexity of interactions among factors mediating post-release opioid-related overdose mortality necessitates coordination across healthcare, criminal justice, and community organizations. The increased prevalence of chronic medical, psychiatric and substance use comorbidities, exposure to stigma, discrimination, and disruption in social networks, and housing instability, unemployment, and poor access to medical care are beyond the scope of a single organization or agency. Further research (Table 1) is needed to determine if care coordination interventions that integrate criminal justice, healthcare and community efforts can reduce post-release opioid-related overdose mortality. Such interventions will need to reduce barriers to social services, facilitate access to health insurance, and reduce interruptions in medical care continuity. Health care and preventative services will need to be tailored to the needs, preferences, and values of PRJP to improve engagement and reduce stigma. Given high rates of prior trauma among those with a history of criminal justice involvement, there is a need for trauma-informed care in primary care and substance use disorder and psychiatric disorder treatment settings. Trauma-informed care has recently been accepted as an important way to address the burden of trauma on health [152, 153]. Programs for formerly incarcerated individuals that have integrated trauma-informed approaches at both the organizational and clinical encounter levels show promise in improving quality of care [154]. Hence, healthcare providers need to be prepared to provide services sensitive to adults released from jail or prison without stigma or discrimination. Further, community partnerships could assist PRJP to integrate back into society and assist with housing and job placement. Harm reduction strategies, such as safe injection sites, and non-opioid pain interventions should also be tailored to the needs of PRJP.

The Post-Release Opioid-Related Overdose Risk Model highlights pathways leading from incarceration to increased overdose risk, but the approach most likely to reduce overdose risk is to reduce the initial exposure to incarceration itself. The punitive and aggressive war on drugs should be ended. The decriminalization of illicit substance use could reduce stigma while also decreasing criminal justice exposure [155]. Police assisted diversion programs, which aim to deflect individuals away from the criminal justice system at the point of entry and to social services and addiction treatment, should be investigated as a means of preventing criminal justice exposure among populations with substance use and mental health disorders [156]. Further research should also clarify if drug courts are a viable means of reducing the harm associated with criminal justice involvement among people with opioid use disorder who are ineligible or lack access to police diversion programs [157].

Finally, we have presented a proposed model by which mediating and modifying factors increase the risk of post-release opioid-related overdose mortality, but this review also presented areas where gaps in knowledge limit our understanding of opioid-related overdose mortality (Table 1). Most national surveys, such as the National Survey on Drug Use and Health, exclude institutionalized adults inhibiting large scale investigation of opioid-related overdose within this population. Improved surveillance data that additionally capture the experiences of PRJP are critical to allow identification and quantification of negative consequences associated with criminal justice involvement, such as opioid-related overdose, and facilitate identification of associated risk factors. The development of our conceptual model also revealed a particular need for studies examining the relationship between risk factors for post-release opioid-related overdose. We have presented a model of post-release opioid-related overdose mortality following release from jail or prison. How the risk of opioid-related overdose is modified by probation or parole exposure or other types of criminal justice exposure is poorly understood and should be the focus of further research.

## Conclusion

Post-release opioid-related overdose mortality is the leading cause of death among PRJP. This paper explored the underlying setting, intermediate, proximate and biological factors which contribute to the risk of post-release opioid-related overdose mortality. Individuals entering the criminal justice system have greater prevalence of past trauma, chronic pain, medical, psychiatric and substance use conditions. Upon entry into the criminal justice system there is a lack of OAT, the first line of therapy for OUD. Incarceration subsequently disrupts an individual’s social network and connection to medical services. Upon transitioning out of the system without OAT or naloxone, individuals are likely to experience stigma, discrimination, suffer from housing instability, and unemployment. In this setting, relapse to opioid use could lead to fatal overdose, because reductions in opioid use during incarceration result in loss of the protective effect of respiratory tolerance. Mitigating the risk of opioid-related overdose mortality following release will require improved coordination across criminal justice, health, and community organizations. Expanding access to OAT and naloxone around the transition period could prevent overdose. Programs are needed to divert individuals with substance use disorder away from the criminal justice system and into treatment and social services, preventing incarceration exposure.

## Abbreviations

PRJP: people released from jail or prison
OUD: opioid use disorder
MOUD: medications for opioid use disorder
OAT: opioid agonist therapy
HIV: human immunodeficiency virus
